# The Biodiversity of Peter I Island—The Most Remote Island in the World

**DOI:** 10.1002/ece3.71634

**Published:** 2025-11-21

**Authors:** Michelle Jackson, Emmett Clarkin, Conor Ryan, Maya Santangelo, Brent Stephenson, Marylou Blakeslee, Sue Forbes, Rich Kirchner, Alex Searle Pineda, Andy Wolff, Tom Hart

**Affiliations:** ^1^ Department of Biology University of Oxford Oxford UK; ^2^ Somerville College University of Oxford Oxford UK; ^3^ National Geographic‐Lindblad Expeditions Seattle Washington USA; ^4^ Scottish Association for Marine Science Oban Argyll UK; ^5^ Eco‐Vista: Photography & Research Havelock North New Zealand; ^6^ University of Chile Santiago Chile; ^7^ School of Biological and Medical Sciences Oxford Brookes University Oxford UK

**Keywords:** Antarctic, BioBlitz, biomonitoring, diversity, marine

## Abstract

Peter I Island is one of the most isolated and least visited islands on earth; lying within the Antarctic Polar Front but over 420 km from continental Antarctica makes it inherently interesting to study in the context of polar biogeography. First discovered in 1821, it was only landed on in 1929 due to challenging ice conditions. Subsequent landings for scientific exploration have been rare, with the primary focus on geological studies and limited biodiversity assessments. To date, only two dedicated marine biodiversity surveys have been conducted, revealing unexpectedly high diversity but limited taxonomic coverage. We provide a comprehensive species list of the area by compiling all previous records with our own data collected in January 2022. Here, a BioBlitz was conducted on the island and surrounding waters, documenting terrestrial, avian and marine species. We identify 15 species new to the region, including benthic and pelagic fauna, as well as an expanded understanding of cetacean and pinniped presence in the area. Marine SCUBA surveys revealed diverse algal and invertebrate communities, emphasising Peter I Island's unique ecological makeup within the Antarctic marine ecosystem. This study establishes a critical biodiversity baseline for ongoing monitoring as the island faces potential ecological shifts due to global change.

## Graphical abstract

Peter I Island is one of the most isolated and least visited islands on earth; lying within the Antarctic Polar Front but over 420 km from continental Antarctica. Here we assess the biodiversity of the island from both previous records and our own BioBlitz in January 2022. We identify 15 species new to the region, as well as an expanded understanding of cetacean and pinniped presence in the area. This is a critical biodiversity baseline for ongoing monitoring as the island faces potential ecological shifts due to global change.

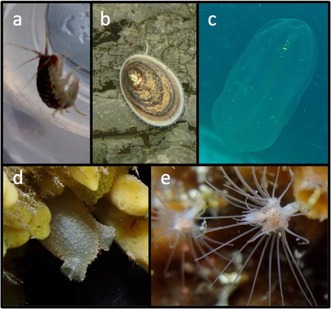

## 1 Background

**FIGURE 1:**
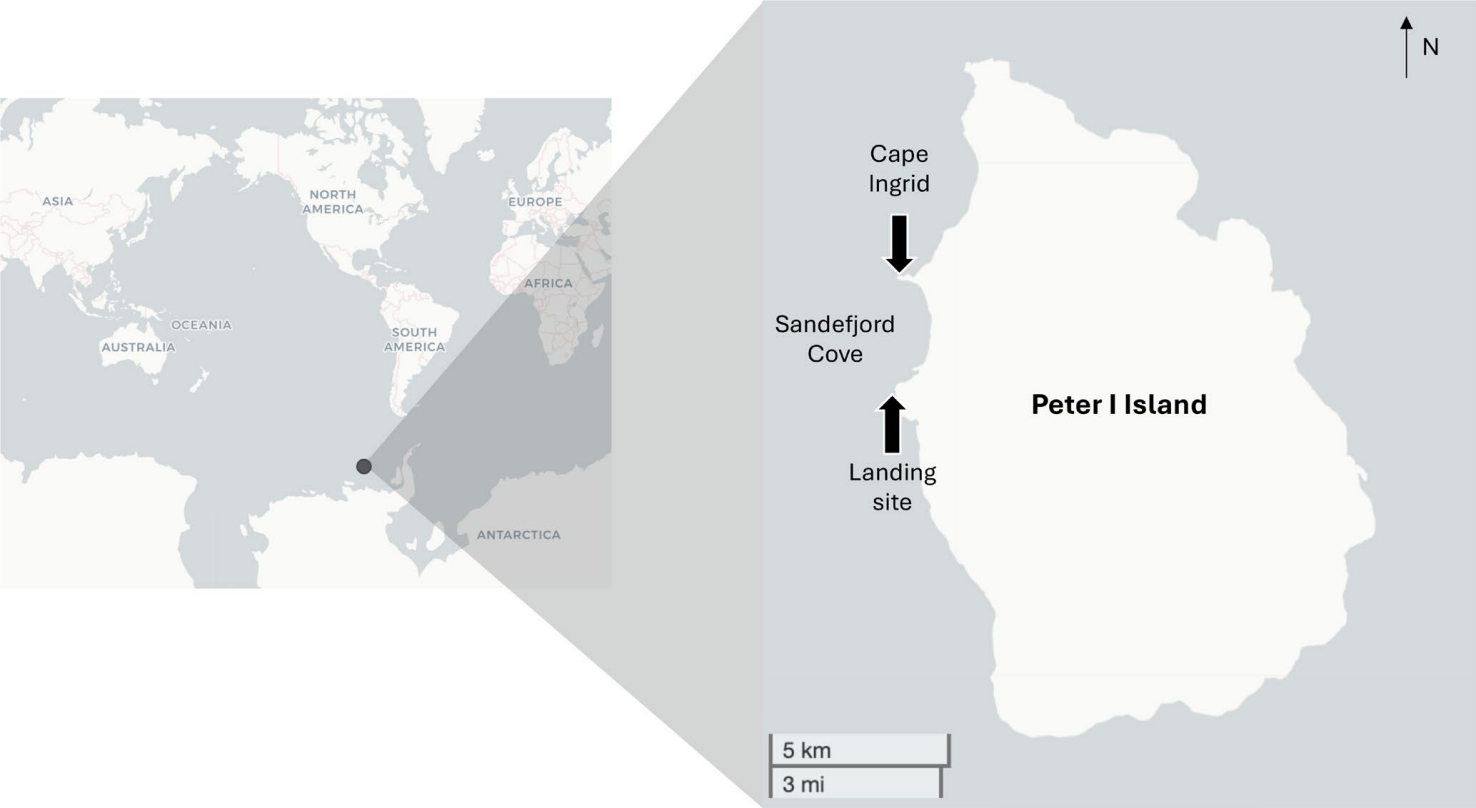
Map of Peter I Island.

Peter I Island (or Peter I Øy) was originally claimed by Norway but now falls within the Antarctic Treaty area (Figure 1). The island of 156km^2^ is almost completely covered by glaciers and despite being first recorded in 1821 (by HIMS Vostok when it was named after Tsar Peter I) it took more than a century before anyone managed to land due to dense pack ice. According to all available evidence, it was not set foot on by humans until 1929, less than 100 years ago (Bulkeley 2013). It was next visited in 1948, and since then only a dozen ships have made the trip for exploration and scientific research. Much of this has been on the geology of this volcanic island (e.g., Prestvik et al. 1990; Hart et al. 1995), and only 2 research cruises have conducted dedicated biodiversity surveys (Troncoso et al. 2007; Matallanas and Olaso 2007; Peña Cantero 2010). These ship‐based surveys present the only scientific record of biodiversity around the island, which is often referred to as the most remote island in the world. Peter I Island has been given this title because of its remote location > 420 km from continental Antarctica (the next nearest landmass) and the major challenges faced when making the journey there (Schalansky 2006).

Even though the few dedicated biodiversity surveys were only conducted from a ship with limited taxonomic coverage, they have indicated extraordinary diversity. For instance, Troncoso et al. (2007) only took four box cores of marine sediment from around the island and found 22 species of molluscs (Table 1), much higher diversity than they discovered in the rest of the Bellingshausen Sea. On the same expedition, Matallanas and Olaso (2007) found nine species of fish in just five traps and one trawl (Table 1). However, these records have never been consolidated in one place, nor are they complete. Therefore, here we collate a comprehensive list of all the species that have been recorded on and around the island using academic papers and historical records. We add our own records from an opportunistic BioBlitz in January 2022. This list should act as a baseline for future surveys as the island begins to be visited more frequently and starts to be altered by climate change.

**TABLE 1:** Species Recorded by Previous Research Trips to Peter I Island and Those Confirmed by Our 2022 BioBlitz in Bold and Underlined. New species records are in bold and highlighted in grey.

**Fishes**	**Molluscs**	**Crustaceans**	**Cnidaria**
*Dissostichus mawsoni*	*Adacnarca nitens*	*Campylaspis excavata*	*Acryptolaria* sp.
*Gobionotothen gibberifrons*	*Adamussium colbecki*	*Cumella australi*	*Antarctoscyphus asymmetricus*
*Lepidonotothen larseni*	*Chlanidota signeyana*	***Euphausia susperba***	*Antarctoscyphus grandis*
*Lepidonotothen squamifrons*	*Cuspidaria infelix*	***Jassa sp***.	*Antarctoscyphus spiralis*
*Notothenia larseni*	*Cuspidaria minima*	*Lithodes macquariae*	*Billardia subrufa*
*Parachaenichthys charcoti*	*Cyamiocardium denticulatum*	*Lithodes murrayi*	*Filellum antarcticum*
*Pogonophryne bellingshausenensis*	*Cyamiomactra laminifera*	***Munna sp***.	*Filellum magnificum*
*Pogonophryne* sp2.	*Cyclopecten notalis*	*Vaunthompsonia inerme*	*Halecium delicatulum*
*Trematomus hansoni*	*Dentalium majorinum*	*Vaunthompsonia laevifrons*	*Halecium frigidum*
*Trematomus scotti*	*Falsilunatia delicatula*		*Halecium pallens*
*Trematomus vicarius*	*Genaxinus debilis*	**Polychaetes**	*Hebella plana*
	*Laternula elliptica*	*Aglaophamus macroura*	*Lafoea dumosa*
**Birds**	*Lissarca miliaris*	*Aglaophamus trissophyllus*	*Lafoea gaussica*
*Daption capensis*	*Lorabela* sp.	*Ammotrypane gymnopyge*	*Schizotricha vervoorti*
***Fulmarsus glacialoides***	*Margarella refulgens*	*Amphicteis gunneri antarctica*	
*Macronectes giganteus*	*Marseniopsis mollis*	*Amphicteis gunneri antarctica*	*Stegella lobata*
***Oceantites oceanicus***	*Marseniopsis syowaensis*	*Aphelochaeta cincinnatus*	*Symplectoscyphus cumberlandicus*
***Pagodroma nivea***	*Melanella antarctica*	*Aricidea belgicae*	*Symplectoscyphus curvatus*
***Pygoscelis adeliae***	*Myonera fragilissima*	*Artacama proboscidea*	*Symplectoscyphus frigidus*
***Pygoscelis antarcticus***	***Nacella concinna***	*Axiothella antarctica*	*Symplectoscyphus glacialis*
***Stercorarius maccormicki***	*Neactaeonina edentula*	*Carinina mawsoni*	*Symplectoscyphus nesioticus*
*Sterna macrura*	*Neobuccinum eatoni*	*Euchone pallida*	*Symplectoscyphus plectilis*
*Thalassoica antarctica*	*Onoba gelida*	*Eulalia subulifera*	*Tubularia antarctica* *Onogorgia nodosa*
	*Onoba kergueleni*	*Galathowenia scotiae*	
**Mammals**	*Onoba turqueti*	*Glycera kerguelensis*	**Other invertebrates**
***Balaenoptera bonaerensis***	*Pareuthria regulus*	*Jasminerira regularis*	*Achelia hoeki*
*Balaenoptera borealis*		*Laetmonice producta*	*Achelia serratipalpis*
*Balaenoptera musculus*	*Philine alata*	*Laonice antacrticae*	*Ammothea calmani*
*Balaenoptera physalus*	*Philobrya sublaevis*	*Laonice weddellia*	*Austroraptus polaris*
*Leptonychotes weddelli*	*Pleurotomella simillima*	*Lubrineris kerguelensis*	***Beroe* sp.**
***Lobodon carcinophaga***	*Probuccinum costatum*	*Maldane srasi antarctica*	*Escharoides tridens*
***Megaptera novaeangliae***	*Probuccinum tenerum*	*Notomastus latericeus*	*Heteronymphon krappi n*.sp.
*Mirounga leonina*	*Prosipho chordatus*	*Ophelina breviata*	*Hippothoa bougainvillei*
*Physeter macrocephalus*	*Prosipho hedleyi*	*Phyllochaetopterus monroi*	*Isodictya filiformis* sp. *nov*.
	*Prosipho pellitus*	*Pista spinifera*	***Liothyrella uva***
**Tunicates**	*Prosipho pusillus*	*Rhodine intermedia*	*Myxilla lissostyla*
***Cnemidocarpa verrucosa***	*Pseudamauropsis anderssoni*	*Spiophanes tcherniai*	*Myxilla pistillaris*
	*Pseudamauropsis aureolutea*	*Thelepus cincinnatus*	*Nymphon multidens*
**Plants and seaweeds**	*Rhabdus cf*. *perceptus*	*Thelepus ehlersi*	*Nymphon nakamurai*
***Desmarestia anceps***	*Siphonodentalium dalli*	*Travisia abyssorum*	*Oligodendrorhynchus hesperides*
***Sarcopeltis skottsbergii***	*Striopulsellum minimum*		*Ophionotus victoriae*
***Iridaea cordata***	*Thyasira bongraini*		*Pentanymphon minutum*
***Myriogramme manginii***	*Trophon cuspidarioides*		*Psilaster charcoti*
***Phycodrys quercifolia***	*Trophon longstaffi*		*Tubulanus mawsoni*
**Plocamium sp**.	*Typhlodaphne innocentia*		***Tubuliporidae idmidronea***
***Prasiola crispa***	*Yoldia eightsii*		
	*Yoldiella antarctica*		
	*Yoldiella profundorum*		

*Note:* Only observations identified to genus or species level are included in this table. References for previous observations: Troncoso et al. (2007), Matallanas and Olaso (2007), Peña Cantero (2010), Mikhalev (2019), Holgersen (1951), Clark (1951), Hartman (1952), Zink (1981), Blanco and Bellusci de Miralles (1972), Klages et al. (1995), Peña Cantero et al. (2002), Troncoso and Aldea (2008), Angel Fernandez‐Alvarez and Anadón Álvarez (2012), Eakin et al. (2008), Corbera et al. (2009), Corbera and Ramos (2005), García Raso et al. (2005), Fernández‐Álvarez and Anadón (2013), Munilla and Soler‐Membrives (2015), Peña Cantero (2010), Parapar et al. (2011, 2013), Ríos and Cristobo (2007), Ríos et al. (2020), Arana and Vega (1999) and Anosov et al. (2015).

## 2 State of Knowledge

Some of the earliest investigations of the marine life around Peter I Island come from whaling activities (Mikhalev 2019). The Discovery Investigations whale marking cruises (from William Scoresby) twice covered parts of the Bellingshausen Sea, ‘almost to Peter I Island’ (Raynor 1940). The closest datapoint to Peter I Island was some 570 km east 68^o^02′ S, 77^o^56′ W (23 January 1938) where a fin whale ( *Balaenoptera physalus* ) was marked and was subsequently caught in the Scotia Sea (Table VII in Raynor 1940). When writing his report in 1940, Raynor describes the area as being ‘unimportant for whaling at the time’. This changed dramatically in the 1960s and 1970s when Soviet whalers focused their efforts in the Bellingshausen Sea including around Peter I Island (Mikhalev 2019). Between 1961 and 1975, during the months December to March, Soviet whaling fleets killed 484 whales within 60 nautical miles of Peter I Island: humpback ( *Megaptera novaeangliae* , *n* = 166), Sei ( *Balaenoptera borealis* , *n* = 116), Antarctic minke ( *Balaenoptera bonaerensis* , *n* = 102), fin (*n* = 61), sperm ( *Physeter macrocephalus* , *n* = 37) and Antarctic blue ( *Balaenoptera musculus intermedia* , *n* = 2; Allison 2020). Whilst the whaling industry was known for its data falsification, particularly in the Soviet whaling fleets (Clapham and Ivashchenko 2009), a summary of whaling research activities by Mikhalev (2019) provides confidence in the statistics quoted above. Humpback whales have also been observed near Peter I Island during visual surveys in 1982, 1989 and 1992 which produced an abundance estimate of 2139 (Branch 2011). The most recent Antarctic minke whale catches in the vicinity of Peter I Island, it appears, were in 1976/1977 (Bushuev and Ivashin 1986).

The earliest species list from Peter I Island was presented at a conference in Uppsala, Sweden, in 1950 by Holger Holgersen (Holgersen 1951) following the Norwegian Brategg expedition. This 2‐year long expedition included the second recorded human landing on the island in 1948 (Bulkeley 2013) which took note of birds, invertebrates, and two species of fish (Holgersen 1951; Table 1). A US Navy expedition was taking place at a similar time in the surrounding area (Clark 1951; Hartman 1952). Many of these early records are corroborated later (e.g., Zink 1981). The next dedicated biological survey was from the Argentinian icebreaker expedition with ARA General San Martín in 1965. This work, along with some other reports which were part of wider Antarctica research (e.g., Klages et al. 1995; Peña Cantero et al. 2002) have since had their results either corroborated or questioned by the more intensive 2003 and 2006 Spanish expeditions on Hespérides. The Spanish expeditions focused on molluscs (e.g., Troncoso et al. 2007; Troncoso and Aldea 2008), but also included fish, cnidaria and other invertebrates (Angel Fernandez‐Alvarez and Anadón Álvarez 2012; Eakin et al. 2008; Corbera et al. 2009; Corbera and Ramos 2005; García Raso et al. 2005; Fernández‐Álvarez and Anadón 2013; Matallanas and Olaso 2007; Munilla and Soler‐Membrives 2015; Peña Cantero 2010; Parapar et al. 2011, 2013; Ríos and Cristobo 2007; Ríos et al. 2020; Table 1), although not all are recorded to species level (Saiz et al. 2013; Moya et al. 2012). Unfortunately, some other surveys could not be included because data was combined for the entire Bellingshausen Sea (e.g., zooplankton studied by Siegel and Harm 1996). Fishery exploration has also confirmed fish records (Arana and Vega 1999) and identified new crabs in the region (Anosov et al. 2015; Table 1). Finally, seaweed has never been investigated in the waters around Peter I Øy (to our knowledge) until our survey. Across the whole of Antarctica, there are 151 known species, with 85 Rhodophyta, 34 Ochrophyta (Phaeophyceae and Chrysophyceae), and 32 Chlorophyta, many of which are endemic to Antarctic waters (Oliveira et al. 2020).

## 3 2022 BioBlitz

We landed on Peter I Island on 5 January 2022 via inflatable boats launched from the expedition cruise ship M/V National Geographic Endurance (operated by Lindblad Expeditions) in the region of Sandefjord Cove, under the command of Captain Aaron Wood. All passengers, scientists and naturalists on board were invited to take part in a BioBlitz, defined as a rapid assessment of biodiversity in a specific area in which multiple people engage and participate (Meeus et al. 2023). Photos were submitted to iNaturalist, and expert Antarctic naturalists on the ship identified organisms. In addition, a dive and a drone team took underwater and aerial footage, respectively, which was later analysed (see below). We report all animal and plant sightings on the island, including those observed on the dive, by drone, from inflatable boats, and the ship upon approach and departure (within 270 km of the island). The purpose of our opportunistic BioBlitz was to add to the state of knowledge for the island. Species missing from our records simply reflect the sampling methods that we used (strictly observational with no trawls, cores or samples taken).

### 3.1 Mammals

**FIGURE 2:**
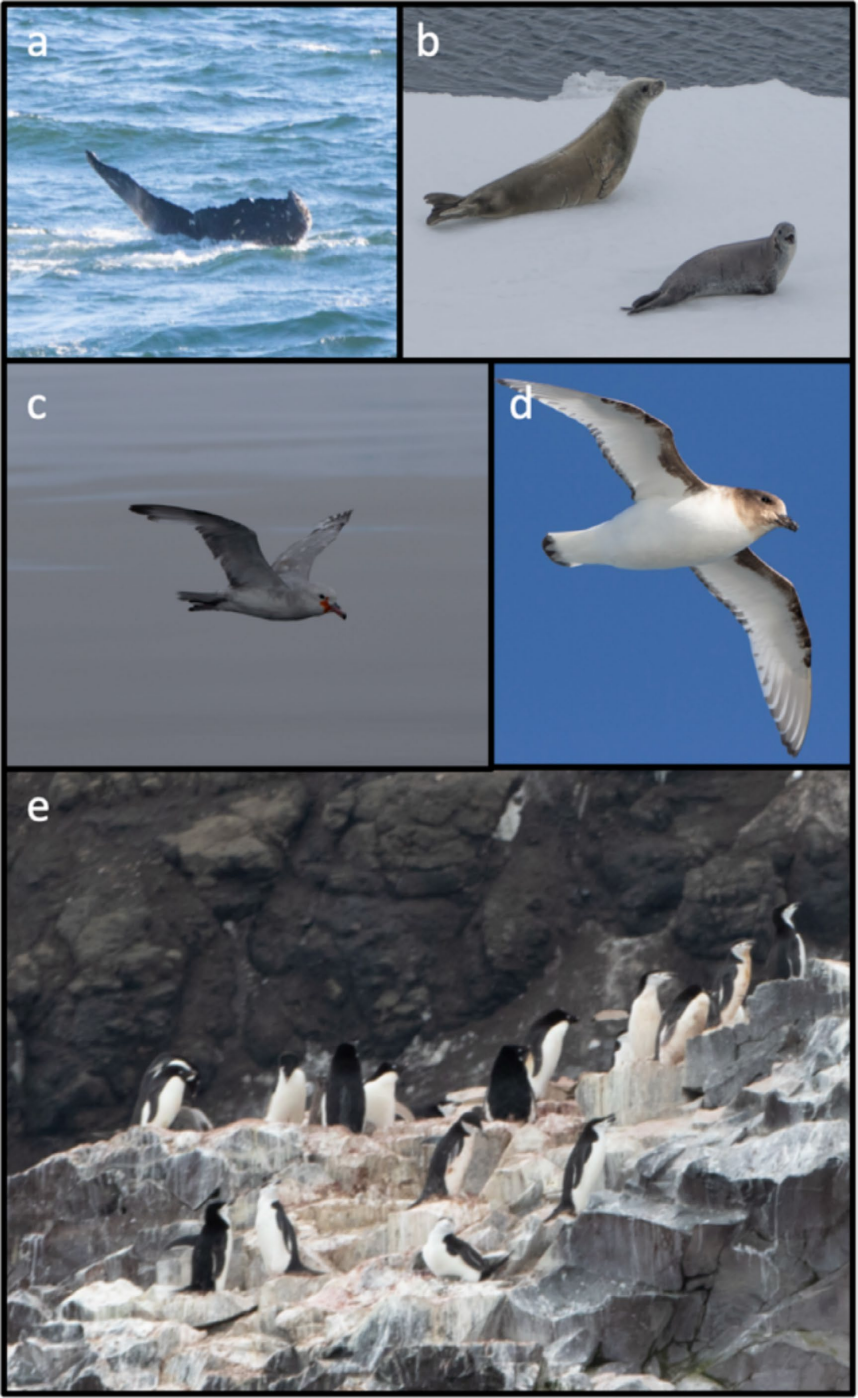
Birds and Mammals around and on Peter I Island. (a) humpback whale, (b) crabeater seals, (c) southern fulmar, (d) Antarctic petrel and (e) nesting chinstrap and adelie penguins. Pictures by CR and MJ.

On 4th, 5th and 6th January 2022, a continuous watch was kept for marine mammals by officers and naturalists on the bridge of the vessel (eye height 12 m above sea level) during daylight hours (Figure 2). Spotting conditions were unusually favourable: partially cloudy, unlimited visibility, no swell, south‐easterly winds (Beaufort scale force 2–5). Bands of pack ice were avoided as much as possible during the transit. Coastal waters were searched for marine mammals in Sandefjord Cove with eight inflatable boats for 2 h on 5th January but remained > 200 m from the shore in places due to dangers from observed rockfalls. Humpback whales were seen on approach in open waters, with the largest aggregation of about 100 individuals spread out over a 40 nautical mile length of the vessel's track, up to the ice edge. On the 6th January, the humpback whale aggregation was encountered in clear water along the margin of a phytoplankton bloom. Antarctic minke whales were observed in more coastal waters. Our observations of humpback and Antarctic minke whales are in keeping with whaling data in suggesting that these are the most common species in the area. Given the deep waters and seamounts near Peter I Island, it is likely that beaked whales such as Arnoux's beaked whale ( *Berardius arnuxii* ) occur but these were not seen; the nearest record is 500 km to the east (Feij et al. 2024).

Information about pinnipeds on or around Peter I Island is very sparse (Bornemann et al. 2000; LaRue et al. 2021). The only species observed in 2022 were crabeater seals ( *Lobodon carcinophaga* ), which were abundant (Table 2). On approach to the island in the northeast, many crabeater seals were hauled out on icefloes in both deep and coastal waters. Upon departure, an aggregation of crabeater seals with an estimated 12 mother and pup pairs was observed on loose pack‐ice at the continental shelf‐edge of the Ronne Entrance 270 km south‐east of Peter I Island. No other species of cetacean or pinniped were observed, despite excellent viewing conditions. However, southern elephant seals ( *Mirounga leonina* ) are known to haul out on a beach to the north of Tsarsporten (Headland 2007).

**TABLE 2:** Marine Mammals observed in the waters around Peter I Island on approach and departure from the Island in January 2022.

Species	Group size (*n*)	Date	Time (GMT)	Latitude	Longitude	Distance from Peter I (km)
Humpback	3	04‐Jan‐22	14:00	−68.1482	−84.2885	260
Humpback	3	04‐Jan‐22	23:32	−68.1895	−84.8308	240
Humpback	~100	05‐Jan‐22	10:00	−68.5657	−89.1094	50
Humpback	~25	06‐Jan‐22	00:00	−68.4478	−88.3459	90
Humpback	10	06‐Jan‐22	01:00	−68.4126	−88.0353	100
Antarctic minke	2	05‐Jan‐22	14:15	−68.6995	−90.9665	10
Crabeater	~50	05‐Jan‐22	11:00	−68.6116	−90.0317	25
Crabeater	6	05‐Jan‐22	16:49	−68.8122	−90.7419	0.1
Crabeater	24	06‐Jan‐22	16:00	−69.8200	−83.8900	270

### 3.2 Birds

We observed seven species of birds during the approach to the island and the bird surveys conducted on 5 January 2022 (Table 3, Figure 2). On the approach we observed three Wilson's storm‐petrel ( *Oceanites oceanicus* ), three Southern fulmar ( *Fulmarus glacialoides* ) and one snow petrel ( *Pagodroma nivea* ). Once at the island, we explored Sandefjord Cove, and found a small rocky promontory which we could land at (tentatively Framnaesodden on some charts). Here, there was a small penguin colony which has been recorded previously. A survey of the colony was made from a vantage point approximately 5–10 m from most of the colony, with approximately 14 active Adelie penguin ( *Pygoscelis adeliae* ) and 28 active Chinstrap penguins ( *Pygoscelis antarcticus* ) nests observed.

**TABLE 3:** Birds observed around and on Peter I Island in January 2022.

Species	Observation notes
South Polar skua	2 birds observed together
Antarctic tern	A single bird seen twice during the surveys showing aggressive defence against the skua pair, possibly indicating breeding behaviour
Adelie penguin	14 pairs at landing site
Chinstrap penguin	28 pairs at landing site
Wilson's storm petrel	Several seen, but only a single bird near the island
Southern fulmar	3500–4500 pairs breeding mainly on Cape Ingrid
Snow Petrel	Approximately 250 pairs estimated for the coastline surveyed. Some pairs found nesting in crevices above the penguin colony (chicks heard beneath brooding adults), and in cliffs nearby. More pairs circling and seen landing around Cape Ingrid

After midday we conducted a further survey of the coastline in Sandefjord Cove, from Framnaesodden to the northern side of Kapp Ingrid, by inflatable. This took approximately 70 min to cover this small area of coastline, making observations with binoculars and doing several drone flights to survey the coastline. Conditions were calm, with relatively clear skies and very good viewing conditions, with almost no breeze. We observed a further two species of bird: two pairs of South Polar skua ( *Stercorarius maccormicki* ) and a single Antarctic tern (*Sterna vittate*; Table 3). Direct evidence of breeding was also found for Southern fulmar and snow petrels (Table 3). Numbers of Southern fulmar were estimated by counting 100 birds on the cliffs and then extrapolating over the area surveyed. Not all birds were likely to be on nests, and some pairs were observed at nest sites, so an overall estimate of 3500–4500 breeding pairs was arrived at. This is likely to be an underestimate, as were the numbers of snow petrels recorded. Snow petrels chose breeding sites that are often hidden in cliffs and, therefore, many may have gone undetected during the survey.

### 3.3 Invertebrates and Plants on Land

Invertebrates and plants observed on the island itself were photographed and identified during our 2‐h landing on 5 January 2022. We were only able to explore ~1800 m^2^ at our landing site (Figure 1) before cliff, ice or sea barriers. Biodiversity was low—we observed the terrestrial algae *Prasiola crispa* and two intertidal invertebrates, an amphipod (family Ischyroceridae) and the limpet *Nacella concinna*. Surprisingly, this is the first record of this limpet at Peter I Island. A past mollusc survey (Troncoso et al. 2007) sampled soft sediment in deeper water using box cores, so it likely missed the shallower hard substrate preferred by limpets.

### 3.4 Underwater Survey

**FIGURE 3:**
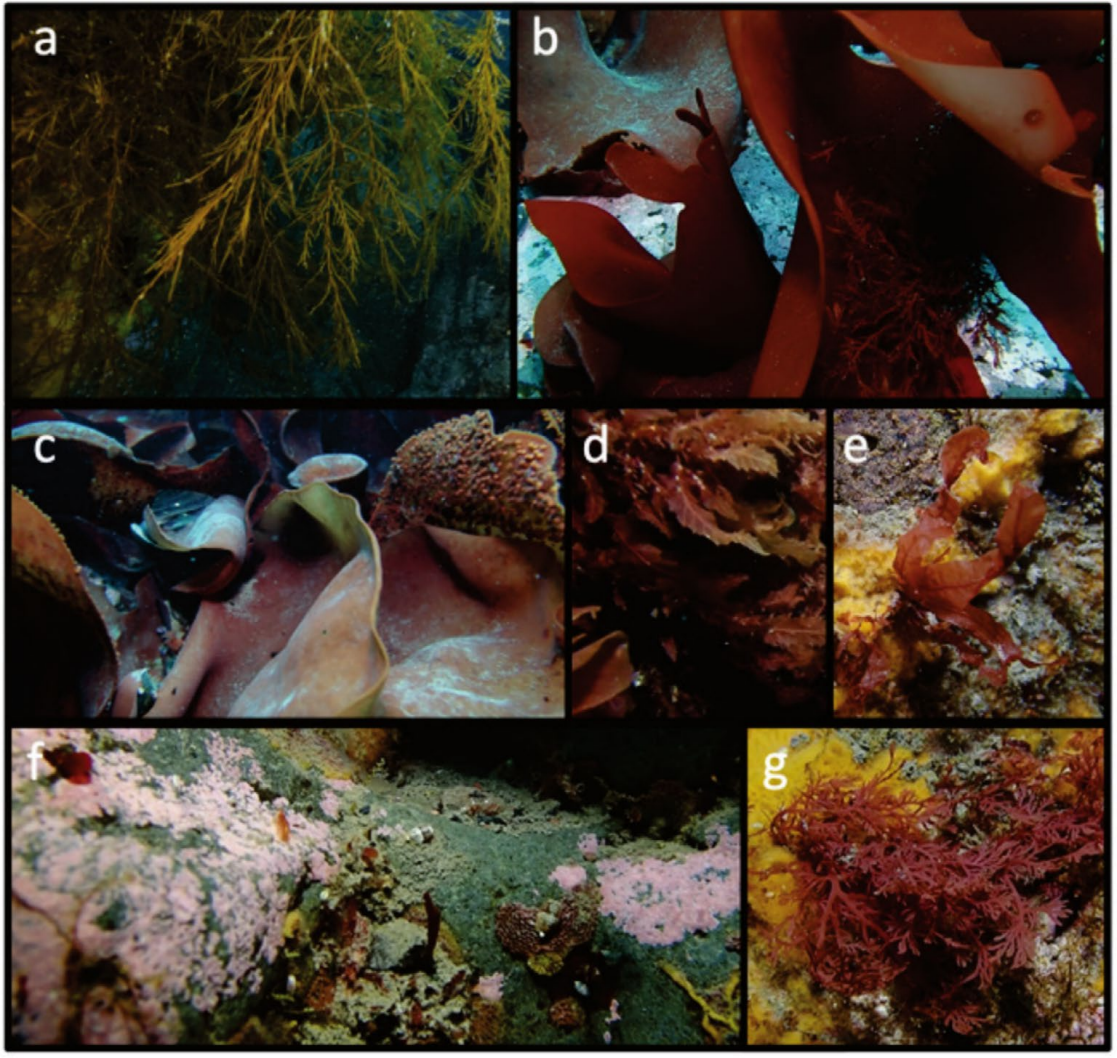
Seaweed at Cape Ingrid. (a) *Desmarestia anceps*, (b) *Iridaea cordata* , (c) *Sarcopeltis skottsbergii*, (d) *Phycodrys quercifolia* , (e) *Myriogramme manginii*, (f) Corallinales, (g) *Plocamium* sp. Pictures by MS.

**FIGURE 4:**
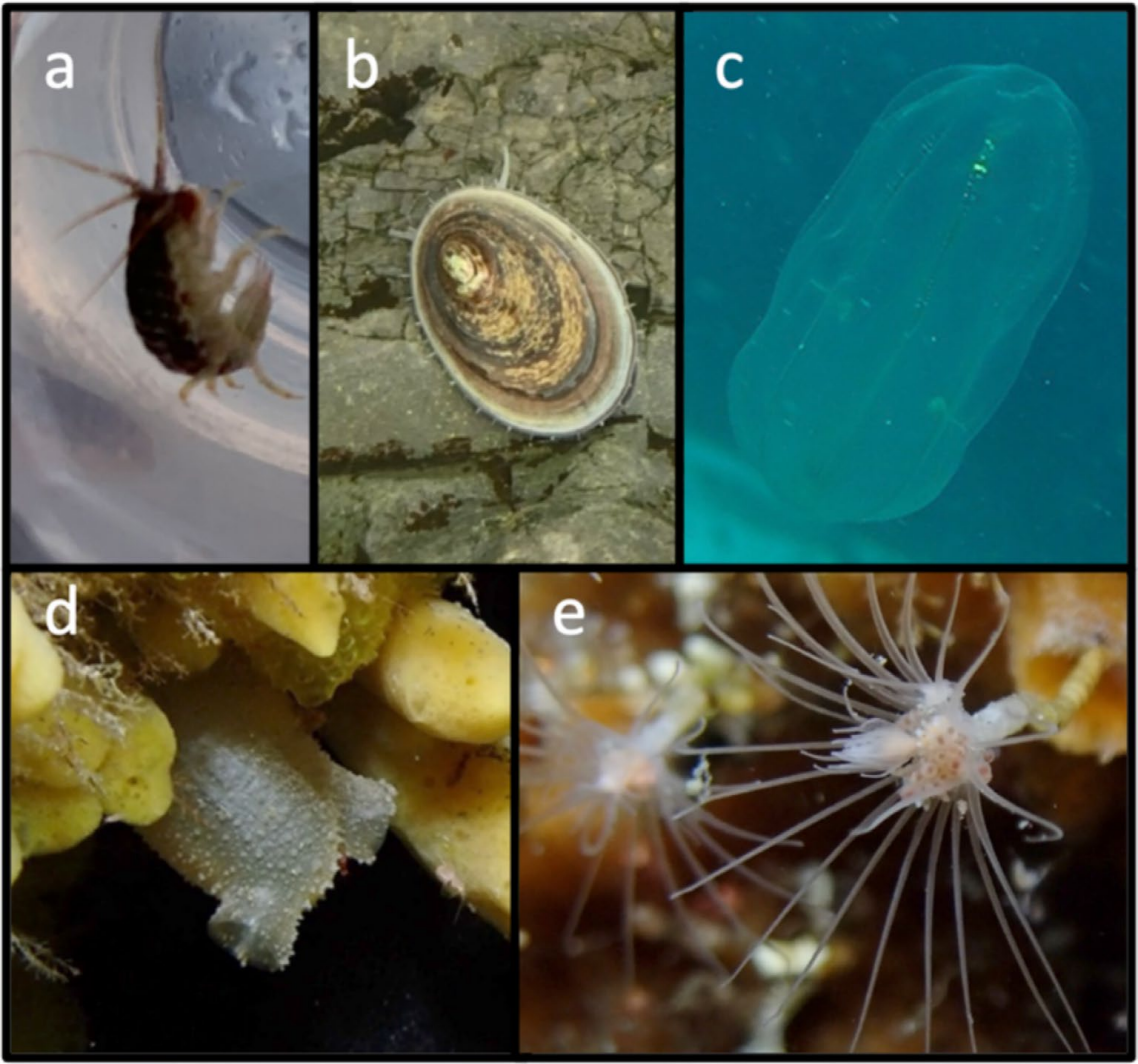
Invertebrates of Peter I Island. (a) Ischyroceridae, (b) *Nacella concinna*, (c) *Beroe* sp., (d) *Cnemidocarpa verrucosa* and (e) *Tubularia antarctica*. Pictures by MS and MJ.

On the 5th January 2022, a SCUBA dive at Cape Ingrid on the northwestern side of Peter I Island took place (Figure 1). The dive was to a maximum depth of 11 m for 36 min, with a water temperature of −2°C and approximately 15 m visibility. The seafloor was a mixture of boulders and rocks, much of which was ice scoured on the upper surfaces, but with many small walls, overhangs, and boulder crevices. We recorded all species using Olympus TG‐6 and Sony RX‐100 mkV cameras with Light & Motion 2500 and 3800 lm video lights attached, respectively. As far as we are aware, no studies have characterised the seaweed diversity of the region before now. We recorded at least seven species (Tables 1 and 4, Figure 3). The most ice‐exposed rocks were covered by crustose red algae in the order Corallinales (Table 1, Figure 3). Red algae were the dominant seaweeds, which is also true for other benthic communities at similar high latitudes (Oliveira et al. 2020). Larger brown seaweeds of the order Desmarestiales were also present on overhangs and within crevices (Table 1, Figure 3); these are known to be dominant in the benthic communities of western Antarctica, growing in zones below that of ice scouring (Wiencke and Clayton 2002). For invertebrates and fish, it was only possible to identify the images to order level in many cases, but these include several orders never recorded in this area before, including Nudibranchia (Table 4, Figure 4). Genus or species level identification was possible for the brachiopod *Liothyrella uva*, the Cetenophore *Beroe* sp., the tunicate *Cnemidocarpa verrucosa*, the *Bryozoan Idmidronea* sp., the Hydroid *Tubularia antarctica*, the amphipod *Jassa* sp. and the isopod *Munna* sp. This is the first record of the phyla Brachiopoda and Ctenophora, subphylum Tunicata, subclass Patellogastropoda, and orders Amphipoda, Isopoda, and Nudibranchia from the water surrounding Peter I Island.

**TABLE 4:** Underwater species list from a single dive in January 2022.

Group	Level of ID	Species
Brachiopoda	Species	*Liothyrella uva*
Bryozoa	Species	*Tubuliporidae idmidronea*
Crustacea (Amphipoda)	Genus	*Jassa* sp.
Crustacea (Isopoda)	Genus	*Munna* sp.
Ctenophora	Genus	*Beroe* sp.
Ctenophora	Order	Lobata
Hydrozoa	Species	*Tubularia antarctica*
Hydrozoa	Family	Candelabridae
Hydrozoa	Order	Leptothecata
Mollusca	Family	Littorinidae
Mollusca	Order (superfamily)	Nudibranchia (Fionoidea)
Mollusca	Order (superfamily)	Nudibranchia (Tritoniodea)
Mollusca	Species	*Nacella concinna*
Porifera	Family	Myxillidae
Porifera	Class	Demospongiae
Tunicata	Order	Aplousobranchia
Tunicata	Species	*Cnemidocarpa verrucosa*
Vertebrata	Family	Nototheniidae
Ochrophyta	Species	*Desmarestia anceps*
Ochrophyta	Species	*Iridaea cordata*
Ochrophyta	Species	*Sarcopeltis skottsbergii*
Ochrophyta	Species	*Phycodrys quercifolia*
Ochrophyta	Species	*Myriogramme manginii*
Ochrophyta	Genus	*Plocamium* sp.
Ochrophyta	Order	Corallinales

## 4 Future Directions

By compiling existing records and doing a new survey, we have established a critical biodiversity baseline for ongoing monitoring of Peter I Island. The new species records that we have added are unlikely to represent range shifts. Instead, they reflect that there has been so little research done in this area before, so these species were simply missed in past surveys. To continue adding to our baseline list, which is unlikely to be complete, future research should use a range of sampling methods, including using environmental DNA to detect species known elsewhere from Antarctica. Creating a comprehensive species list is especially important in the face of global change. It is critical that we establish what biodiversity is present in remote locations before it starts to change with global warming and other anthropogenic pressures, including pollution and species invasions. Future research should expand the existing species list, continue to monitor the biodiversity of the island over time, and establish more accurate records of the abundance of key species—particularly those that are declining elsewhere due to avian influenza (subtype H5N1; Brownell Jr et al. 2024). This could be done with more targeted drone surveys and transects (both above and below the ocean surface). It would also be important to collect samples for population genetics of the species, as some might be unique due to the remote location of that island.

Since we completed this survey, nine more cruise ships have visited the area (although only three have managed to land on the island, according to International Association of Antarctica Tour Operators), so there is potential to continue monitoring using this rapid and light‐touch approach. However, this also raises concerns about the impacts visitors to the region might have by disturbance. The International Association of Antarctica Tour Operators sets out some useful guidance for the practise of safe and environmentally responsible travel in the region which should always be followed.

## Author Contributions

**Michelle Jackson:** conceptualization (lead), data curation (equal), investigation (equal), methodology (equal), project administration (equal), resources (equal), supervision (lead), validation (lead), visualization (equal), writing – original draft (lead). **Emmett Clarkin:** data curation (equal), investigation (equal), methodology (equal), visualization (equal), writing – review and editing (supporting). **Conor Ryan:** data curation (equal), investigation (equal), methodology (equal), visualization (equal), writing – review and editing (supporting). **Maya Santangelo:** data curation (equal), investigation (equal), methodology (equal), visualization (equal), writing – review and editing (supporting). **Brent Stephenson:** data curation (equal), investigation (equal), methodology (equal), resources (equal), writing – review and editing (supporting). **Marylou Blakeslee:** data curation (equal). **Alex Searle Pineda:** data curation (equal). **Sue Forbes:** data curation (equal). **Rich Kirchner:** data curation (equal). **Andy Wolff:** data curation (equal). **Tom Hart:** data curation (equal), investigation (equal), methodology (equal), resources (equal), writing – review and editing (supporting).

## Conflicts of Interest

The authors declare no conflicts of interest.

## Data Availability

All data is provided in tables in the paper.
